# BUTIMBA: Intensifying the Hunt for Child TB in Swaziland through Household Contact Tracing

**DOI:** 10.1371/journal.pone.0169769

**Published:** 2017-01-20

**Authors:** Anna Maria Mandalakas, Katherine Ngo, Pilar Alonso Ustero, Rachel Golin, Florence Anabwani, Bulisile Mzileni, Welile Sikhondze, Robert Stevens

**Affiliations:** 1 The Global Tuberculosis Program, Texas Children's Hospital, Section of Global and Immigrant Health, Department of Pediatrics, Baylor College of Medicine, Houston, United States of America; 2 Baylor College of Medicine Children's Foundation—Swaziland, Mbabane, Swaziland; 3 Swaziland National Tuberculosis Control Program, Mbabane, Swaziland; 4 Mott McDonald, London, United Kingdom; McGill University, CANADA

## Abstract

**Background:**

Limited data exists to inform contact tracing guidelines in children and HIV-affected populations. We evaluated the yield and additionality of household contact and source case investigations in Swaziland, a TB/HIV high-burden setting, while prioritizing identification of childhood TB.

**Methods:**

In partnership with 7 local TB clinics, we implemented standardized contact tracing of index cases (IC) receiving TB treatment. Prioritizing child contacts and HIV-affected households, screening officers screened contacts for TB symptoms and to identify risk factors associated with TB. We ascertained factors moderating the yield of contact tracing and measured the impact of our program by additional notifications.

**Results:**

From March 2013 to November 2015, 3,258 ICs (54% bacteriologically confirmed; 70% HIV-infected; 85% adults) were enrolled leading to evaluation of 12,175 contacts (median age 18 years, IQR 24–42; 45% children; 9% HIV-infected). Among contacts, 196 TB cases (56% bacteriologically confirmed) were diagnosed resulting in a program yield of 1.6% for all forms of TB. The number needed to screen (NNS) to identify a bacteriologically confirmed TB case or all forms TB case traced from a child IC <5 years was respectively 62% and 40% greater than the NNS for tracing from an adult IC. In year one, we demonstrated a 32% increase in detection of bacteriologically confirmed child TB. Contacts were more likely to have TB if <5 years (OR = 2.0), HIV-infected (OR = 4.9), reporting ≥1 TB symptoms (OR = 7.7), and sharing a bed (OR = 1.7) or home (OR = 1.4) with the IC. There was a 1.4 fold increased chance of detecting a TB case in households known to be HIV-affected.

**Conclusion:**

Contact tracing prioritizing children is not only feasible in a TB/HIV high-burden setting but contributes to overall case detection. Our findings support WHO guidelines prioritizing contact tracing among children and HIV-infected populations while highlighting potential to integrate TB and HIV case finding.

## Introduction

Tuberculosis (TB) is the leading infectious cause of morbidity and mortality worldwide[1]. In 2015, an estimated 1.4 million people died of TB, including 0.4 million HIV-infected and 210,000 children. In that same year, 10.4 million people developed TB including 1.2 million people living with HIV and 1.0 million children. Although 6 million new cases of TB were reported to the World Health Organization (WHO), 41% of new cases went undiagnosed or were not reported. To reduce the global burden of TB, this detection gap must be closed.

Case finding and treatment of TB disease are central to controlling transmission and reducing incidence [2]. Although historically TB high burden settings have been reliant on passive self-presentation of symptomatic individuals (passive case finding), active case finding (ACF) has long been considered the cornerstone of TB control in many TB low burden settings. ACF strategies range from population wide screening to targeted case-finding in high risk groups such as people living with HIV and contacts of known TB cases [3–5]. In 2012, the WHO released recommendations to guide the investigation of TB contacts in middle and high-burden settings [5]. Contact investigations evaluate contacts of an infectious adult or adolescent for TB or LTBI and often focus on household contacts. Source case investigations evaluate all contacts of a child with TB to identify an infectious adult or adolescent and may have lower yield than contact investigation but evidence is lacking. Two meta-analyses of contact investigations found a pooled prevalence of all form of active TB among close contacts to be 3.1% (95%CI 2.2–4.4) and 4.5% (95%CI 4.3–4.8); pooled prevalence of microbiologically confirmed TB was 1.2% (95% CI 0.9–1.8) and 2.3% (95% CI 2.1–2.5)[6, 7]. Nevertheless, these systematic reviews demonstrated strong heterogeneity in the yield of contact investigations and suggested a number of factors that might influence yield including characteristics of the study setting, index cases, and contacts.

Fueled by the Stop TB Partnership TB REACH initiative, innovative approaches to ACF have increased among populations thought to have increased risk of developing TB across a spectrum of settings [8]. Meta-analysis of these recent initiatives demonstrated that program yield and contribution to case detection are dependent on program design and implementation [9]. Eligibility criteria used to guide evaluation of contacts demonstrated that compared to the most restrictive criteria (≥ 2 weeks of cough) the adjusted odds ratio for lesser (any TB related symptom) and the least (all contacts) restrictive testing criteria were 1.71 (95% CI 0.94–3.13) and 6.90 (95% CI 3.42–13.93), respectively [9]. Furthermore, contact investigation contributed to between <1% to 14% of all sputum smear positive cases in the intervention areas.

Although recommended by the WHO and Swaziland’s National TB Control Programme (NTCP) for over a decade, community-based ACF was implemented in Swaziland in 2016. The delay in implementation was presumably due to lack of training, supportive tools, limited accessibility to lab services, and high patient burdens [10]. Supported by the TB REACH initiative, we introduced household contact and source case investigation in three of Swaziland’s four regions. Previous ACF studies focused solely on the impact, feasibility and sustainability of this intervention on increasing TB case detection rates in Swazi adults [10, 11]. Despite numerous peer-reviewed articles documenting that household proximity to a bacteriologically confirmed adult TB is an important predictor of a positive TB screen in children, there is very little published data on ACF interventions targeting childhood TB in TB high-burden, resource-limited settings [12, 13]. Gaps in the literature regarding the childhood TB diagnostic cascade include limited evidence to inform standardized childhood TB contact tracing algorithms or to improve the diagnosis of paucibacillary TB in children [14]. As there is little evidence available regarding the yield of household contact and source case investigations among children in TB/HIV high burden settings, the objective of our study was to evaluate the yield and additionality of these strategies in a TB/HIV high burden setting while prioritizing the identification of TB among child contacts.

## Materials and Methods

In 2013, Baylor College of Medicine Children’s Foundation–Swaziland (BCMCF-SD) received funding from Stop TB Partnership’s TB REACH Wave 3 initiative to increase TB case detection in Swaziland. This project, aptly named “Butimba” (meaning “The Royal Hunt” in siSwati), prioritized TB diagnosis and prevention in children (< 15 years old of age) living in a TB/HIV high burden setting.

**Fig 1:**
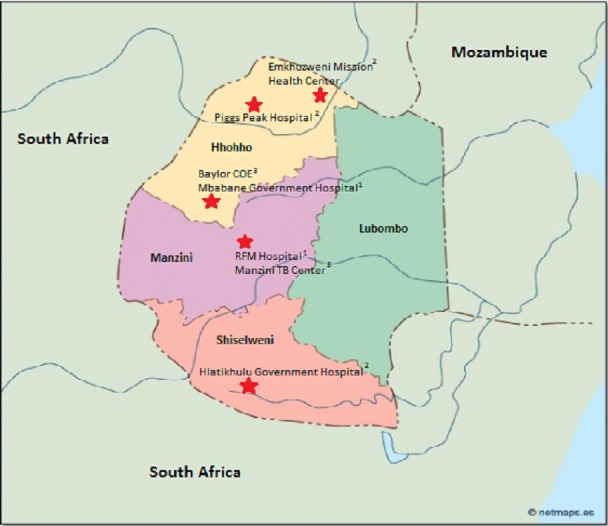
Basic Management Units in Swaziland supported by Wave 3 TB Reach. ^1^Tertiary level; National or Regional Referral Hospital. ^2^Secondary level; Regional Hospital or Health Center. ^3^Primary level; Referred Specialized Care Health Center.

The Butimba project implemented active case finding in seven Basic Management Units (BMUs) in three of Swaziland’s four regions. (Fig 1) Partnering BMUs included one national referral hospital, three district hospitals, two referral clinics and one rural health centre. To capture difficult-to-reach populations and to ensure programmatic implementation success, BMUs were chosen based on patient volume, setting, and existing institutional relationships.

Lay screening officers were assigned to each partnering BMU to perform household contact tracing of index cases starting anti-TB treatment (ATT) and refer eligible contacts for Isoniazid Preventive Therapy (IPT). In addition, screening officers collected and transported expectorated sputum, but did not complete sputum induction or gastric aspiration on young children. Screening officers at BCMCF-SD family-centered HIV clinic also performed TB screening of clients when they presented for routine care, and referred eligible clients for initiation of IPT as part of a comprehensive HIV care package. Screening officers’ education level, training, and role on the health care team were typical of programmatic standards within Swaziland. Upon hire, screening officers completed a four day intensive training required by the national TB program. In addition, screening officers completed training of program protocols, data capture, good clinical practice, and child and adult TB. Screening officers received daily support from peer screening officers and nurse clinicians employed by the Ministry of Health at participating program sites and supervision every other week by senior clinicians from our Butimba program team.

Screening Officers utilized a project-specific Family Mapping Tool (FMT) to obtain information regarding each reported member of an index case’s household (S1 File). Screening Officers interviewed index cases to enumerate households and gather information regarding household contacts using an age stratified approach. Information gathered included age, gender, HIV status, sleep location, relationship to index case and presence of symptoms consistent with TB. TB symptom screening was consistent with NTCP guidelines. Screening officers instructed the index cases to return to the BMU with household contacts who were reported TB symptom screen positive for further clinical evaluation of presumptive TB. Contacts that had a negative TB screen and who qualified for IPT were also invited via the index case to return to the BMU for IPT initiation. A concentrated effort was made to encourage index cases to return with child contacts under five years of age.

After obtaining the index case’s permission, screening officers visited homesteads whose contacts with presumptive TB did not return to the BMU within 2 weeks of FMT completion on average. Homesteads eligible for visits were prioritized if 1) the associated index case had bacteriologically confirmed TB (smear, GXP, and/or culture positive), 2) household contacts included children, or 3) multiple contacts reported to have a positive TB symptom screen. During the home visits, screening verified the FMT information provided by the index case and added any newly identified contact to the FMT. Home visits were scheduled on Mondays through Saturdays to meet the scheduling needs of contacts that had school or work obligations during the work week.

Prior to arrival, screening officers verified which contacts would be present during the home visit. Contacts with presumptive TB who would not be present were asked to leave an early morning sputum specimen for the screening officers to collect. After providing instructions to optimize expectoration, screening officers collected sputum from household contacts who were able to expectorate and delivered these to the clinic before close of business. Contacts who were unable to expectorate sputum were referred to the respective BMUs for an alternative sputum collection technique such as gastric or nasopharyngeal aspiration or sputum induction. Contacts referred to a BMU were given a “fast track” card to shorten their wait time and potential for secondary spread of TB to others.

Each facility sent samples to their respective laboratory for routine processing according to routine practice. Samples were processed within 24 to 48 hours of collection depending on protocol at the facility. All participating laboratories were part of the national laboratory system which provides accreditation and quality assurance monitoring. On days when sputum collection was particularly robust and overwhelmed the routine capacity, overflow specimens would be processed at the Baylor clinic TB laboratory which is also accredited and monitored by the national laboratory system.

BMU clinicians evaluated, diagnosed, and treated contacts for TB. They also initiated IPT for eligible household contacts, prioritizing children under the age of 5 years. Six of seven BMUs had on-site radiography available. The seventh BMU, the main BCMCF-SD clinic, initially referred clients to the national referral hospital for radiography but later acquired on-site capacity. Although the rural health centre BMU utilized only AFB smears for the first five months of the project, by the end of the project all of the sites used GeneXpert as the initial diagnostic test. Numerous barriers to accessing healthcare in Swaziland exist; some of these barriers include transport fare, facility fees, and x-ray fees. To help decrease these obstacles, Butimba contacts were provided with transport fare reimbursement and vouchers for free chest x-rays.

Following standardized procedures, screening officers regularly entered the FMT data into a Data Extraction Tool (DET); the DET also captured sputum laboratory and radiography results and data regarding IPT uptake extracted from clinic records and registers. At routine intervals, data officers would enter the DET data into a web-based database and conduct quality assurance checks. Quality assurance and data cleaning sessions were convened with the Screening Officers on a quarterly basis.

Program data was entered into a web-based database developed and secured by the Baylor College of Medicine’s Dan L. Duncan Institute for Clinical and Translational Research Center (ICTR) (Houston, TX), and data analysis was performed using STATA version 11.2 for Windows (StataCorp LP, College Station, Houston, TX, USA).

Summary statistics were performed to compare the characteristics between Index Cases and Contacts as well as between those who had disease and no disease using Chi Square analysis and Student’s t test. After this initial analysis, variables with a p value < 0.05 were included in the multinomial logistic regression model. Multinomial logistic regression modeling was utilized to infer disease status within and across multiple categorical variables of interest. To address household size and to account for within cluster correlation of variable effects on contact disease diagnosis, cluster analysis was performed using the Generalized Estimating Equations (GEE) method.

Percent yield is defined as 100 times the total number of all forms of TB cases found divided by the total number of contacts screened for TB. The Number Needed to Screen (NNS) is calculated as 100 divided by the percent yield [9].

Program impact was also assessed by additional notifications, which are a programmatic and pragmatic measure that explicitly includes improvements in surveillance data quality in its definition [15]. This method of ongoing outcome monitoring compared the number of health events recorded in project data to population-level registers and allowed recording errors and registrations outside the six BMU evaluation population to be investigated, and variations between project sites to be explored against an independent data source. Additional notifications were regression-adjusted for baseline trend to account for declining TB case notification rate in the study setting. Interpretation of additionality considered factors external to the project that could affect surveillance data.

All clinical investigation supporting the reporting of these findings was conducted according to the principles expressed in the Declaration of Helsinki. All participants gave oral consent for participation in the Butimba program. No individual participant consent was required for program evaluation as the data were analysed anonymously. Approval was obtained from all necessary ethical bodies including the Baylor College of Medicine Children’s Foundation Swaziland and the Swaziland Ethics Committee (24047712/24045469) in Swaziland, and the Baylor College of Medicine Institutional Review Board (H-35028), Houston, Texas, USA.

## Results

From March 1, 2013 to November 31, 2015, the Butimba project enrolled 1,568 index cases (median age 32.2 years; IQR 23.5–41.7) of whom the majority were bacteriologically confirmed (53.5%), HIV infected (69.9%), and adults (85.4%)(Table 1 and Table 2). Investigation of the index case households led to the evaluation of 12,175 contacts (median age 17.7 years; IQR 23.5–41.7) of whom 44.5% were children and 9% reported HIV-infection; the majority of household members (55.4%) reported unknown HIV status. Of homes reporting a death within the preceding two years (26.1%), TB, HIV and TB/HIV were the cause of mortality 32.5%, 10.1% and 8% of the time respectively. A household member was employed as a miner in 25% of the homes. A small portion of contacts (23.7%; 2,881/12,175) were eligible to receive IPT due to having HIV-infection or being less than 5 years; of these, 8.5% (246/2,881) initiated IPT. Broken down further, of the contacts who were symptom screened negative, 1,806 (25%; 1,806/7,237) were eligible for IPT. Of those eligible, 9.3% (168/1,806) were started on IPT. Moreover, there were contacts that symptom screened positive and subsequently deemed to not have TB disease. From these contacts, 1,075 (23%; 1,075/4,723) were eligible for IPT and 7.3% (78/1,075) were started on IPT.

**Table 1:** Demographic and clinical characteristics of individuals enrolled.

	Index cases		Contacts	
	(N = 3258)		(N = 12175)	
	n	(%)	n	(%)
Female sex	1568	(48)	6951	(57)
Age, years, median [IQR]	32 [24–42]		17.7 [24–42]	
Range	0–82		0–84	
Age group, years	n = 3258		n = 12175	
<5	256	(8)	1944	(16)
5–14	219	(7)	3474	(29)
≥15	2783	(85)	6757	(56)
HIV status	n = 2981		n = 12158	
Positive	2083	(70)	1099	(9)
Negative	703	(24)	4322	(36)
Unknown	195	(7)	6737	(55)
Death in the home in past two years	n = 3220		n = 12081	
Yes	771	(24)	3225	(26)
No	2449	(76)	8856	(74)
Cause of household death	n = 771		n = 3225	
TB	226	(29)	1047	(33)
HIV	87	(11)	327	(10)
TB and HIV	53	(7)	258	(8)
Accident	36	(5)	141	(4)
Other	369	(48)	1452	(45)
Miner in the household	n = 3258		n = 12175	
Yes	737	(23)	3132	(25)
No	2521	(77)	9043	(75)
Sleep location to index case	N/A		n = 12212	
Different house			5354	(44)
Same house			3855	(32)
Same room			2208	(18)
Same bed			795	(7)
Relation to index case	N/A		n = 12175	
Child			2618	(22)
Parent			1323	(11)
Other			8234	(68)
TB screen	N/A		n = 12158	
Positive			4889	(40)
Negative			7269	(60)
Number of TB symptom screen negative eligible for IPT*	N/A		n = 1806	
Number started on IPT			168	(9)
Number of TB symptom screen positive eligible for IPT*	N/A		n = 1075	
Number started on IPT			78	(7)

*Eligible for Isoniazid Preventive Therapy (IPT) defined as contacts with TB disease ruled out with HIV or under 5 years of age

**Table 2:** Result of Sputum Evaluation Among Index Cases and Contacts Reporting TB Symptoms.

	Index cases		Contacts	
	(N = 3258)		(N = 4889)	
	n	(%)	n	(%)
Bacteriologically confirmed*	1744	(54)	109	(22)
Bacteriologically negative	662	(20)	3740	(77)
Unknown bacteriologic status	852	(26)	1040^§^	(21)

*Having either a GeneXpert result of “MTB detected”, sputum smear microscopy result of “sputum smear positive (SS+)”, or “positive” culture result.

^§^ Includes 13 with no results, 1027 specimens not collected

Contacts reporting one or more symptoms consistent with TB (40.2%; 4,889/12,175) were referred for further evaluation at their local TB clinic. An additional 196 TB cases were diagnosed among household contacts of which 55.6% (109/196) were bacteriologically confirmed cases (Table 2). Hence, our program experienced a 1.6% yield for all forms of TB.

TB diseased contacts were more likely to be HIV-infected (p≤0.0001) and sleep in the same bed as the index case (p≤0.0001) while contacts without TB disease were more likely to be 5–14 years of age(p = 0.003), have unknown HIV status (p≤0.0001) and reside in a different home (p≤0.0001). TB diseased contacts were twice as likely to report one or more TB symptoms compared to contacts without TB disease (84.7% and 39.5%, respectively; p<0.0001) (Table 3).

**Table 3:** Demographic and clinical characteristics of contacts by disease status.

	TB Disease		No Disease		
	n	(%)	N	(%)	p-value
Female sex	114	(58)	6824	(57)	0.76
Age, years, median [IQR]	23 [8–34]		18 [8–33]		0.45
Range	0–69		0–84		
Age group, years	n = 196		n = 11960		0.003**
<5	40	(20)	1902	(16)	
5–14	35	(18)	3435	(29)	
≥15	121	(62)	6623	(55)	
HIV status	n = 196		n = 11960		0.000**
Positive	83	(64)	1016	(9.0)	
Negative	61	(22)	4261	(29)	
Unknown	52	(15)	6683	(55)	
Death in the home in past two years	n = 192		n = 11871		0.96
Yes	51	(24)	3173	(27)	
No	141	(76)	8698	(73)	
Cause of household death	n = 51		n = 3173		0.87
TB	16	(29)	1030	(33)	
HIV	7	(11)	320	(10)	
TB and HIV	3	(7)	255	(8)	
Accident	3	(5)	138	(4)	
Other	22	(48)	1430	(45)	
Sleep location to index case	n = 196		n = 11959		0.000**
Different house	67	(34)	5252	(44)	
Same house	39	(20)	752	(6)	
Same room	41	(21)	3804	(32)	
Same bed	49	(25)	2151	(18)	
TB screen	n = 196		n = 11960		0.000**
Positive	166	(85)	4723	(40)	
Negative	30	(15)	7237	(61)	

**Significant at p≤0.01

**Fig 2:**
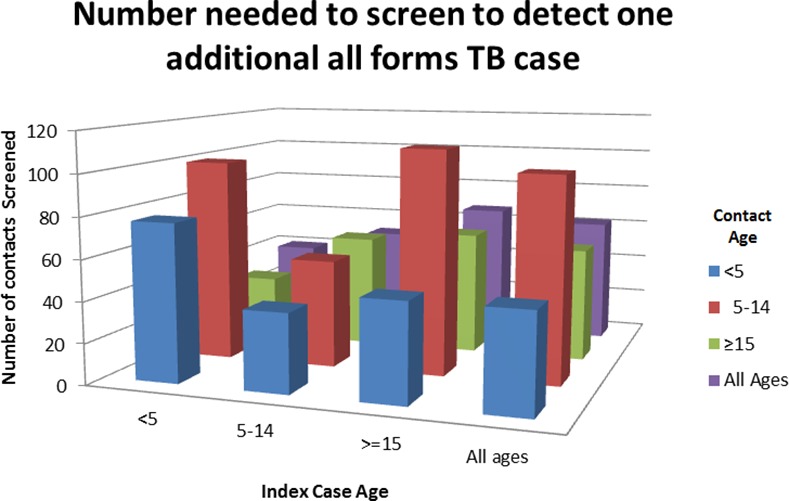
Number needed to screen to detect one additional all forms TB case.

The Butimba program identified 1,609 cases of all forms of TB for every 100,000 contacts screened resulting in a yield of 1.6%. Butimba identified 1,390 cases of all forms of TB for every 100,000 child contacts screened. In comparison, Butimba identified 1,798 cases of all forms of TB for every 100,000 adult contacts screened. The number needed to screen (NNS) to detect one bacteriologically confirmed case or one TB case of any form varied by age of the index case and by age of the contacts (Table 4, Fig 2). The NNS to identify a bacteriologically confirmed TB case traced from a child index case less than five years of age (n = 175) was 62% greater than the NNS for tracing from an adult index case (n = 108). Similarly, the NNS to identify any all forms TB case traced from a child index case less than 5 years of age (n = 40) was 40% greater than the NNS for tracing from an adult index case (n = 67). Among contacts of adult index cases, over five times as many child contacts under five years (n = 417) and twice as many children aged 5–14 years (n = 168) needed to be screened to detect one bacteriologically confirmed pediatric case compared to adult contacts (n = 77). In comparison, to detect any case of TB traced from an adult index case, 48 child contacts, 110 contacts age 5–14 years, and 61 adult contacts age greater than 14 years needed to be screened. NNS to detect a bacteriologically confirmed TB case was more than halved in contacts that provided sputum when compared to NNS in contacts that were only symptom screened (Table 4).

**Table 4:** Number needed to screen to detect one additional TB case.

**Number needed to screen to detect one additional case of all forms of TB**				
	** **	**Contact age (years)**	** **	
**Index case age (years)**	**<5**	**5–14**	**≥15**	**All ages**
** <5**	77	100	31	40
** 5–14**	39	53	56	51
** ≥15**	48	110	61	67
**All ages**	48	100	56	63
Number needed to screen to detect one bacteriologically* **confirmed TB case**				
	** **	**Contact age (years)**	** **	
**Index case age (years)**	**<5**	**5–14**	**≥15**	**All ages**
** <5**	N/A	N/A	100	175
** 5–14**	N/A	77	110	109
** ≥15**	417	168	77	108
**All ages**	476	164	80	112
Number needed to screen to detect one bacteriologically* **confirmed TB case from contacts who provided sputum**				
	** **	**Contact age (years)**	** **	
**Index case age (years)**	**<5**	**5–14**	**≥15**	**All ages**
** <5**	n/a	n/a	24	28
** 5–14**	n/a	22	29	27
** ≥15**	29	51	26	31
**All ages**	48	50	26	32

*Defined as having either a GeneXpert result of “MTB detected”, positive sputum smear microscopy (SS+), or positive culture result.

During the first year of the program, Swaziland shows a steady decrease in the case notification rate of TB at national level. Nevertheless, our program demonstrated a 32% increase in case detection of bacteriologically confirmed children, with an adjusted additionally of 138 child TB cases. Similar analysis was completed to measure additionality in year two and demonstrated smaller but positive additionality for all forms of TB in children, with an adjusted additionality of 64 child TB cases.

Multivariate regression analysis controlled for characteristics of the contact (age, HIV status, presence of TB symptoms, sleep proximity to index case) and the presence of an HIV-infected person in the household. (Table 5) Similar to bivariate analysis, contacts were more likely to have TB disease if they were younger than 5 years of age (OR = 2.01), reported HIV-infection (OR = 4.9), reported one or more TB symptoms (OR = 7.70), shared a bed with the index case (OR = 1.65) or lived in a home affected by HIV (OR = 1.44). In contrast, contacts with unknown HIV status were less likely to have TB disease (OR = 0.63).

**Table 5:** Association of Contact Characteristics and TB Diagnosis.

Characteristic	OR	95% CI	P Value
**Age group 0–4.99 years**	2.01	1.26–3.21	0.004
(Referent Group: Age group 5–14)			
**Age group ≥15 years**	1.17	0.79–1.74	0.425
**Reported Positive HIV Status**	4.93	3.41–7.13	<0.001
(Referent Group: HIV Negative)			
**Reported Unknown HIV Status**	0.63	0.43–0.92	0.017
**Positive TB Symptom Screen**	7.70	5.16–11.47	< .0001
(Referent Group: TB Symptom Screen Negative)			
**Sleep in same house as Index Case**	0.72	0.48–1.07	0.109
(Referent Group: Sleep in different house)			
**Sleep in same room as Index Case**	1.28	0.87–1.89	0.214
**Sleep in same bed as Index Case**	1.65	1.07–2.54	0.024
**HIV affected Household**	1.44	1.06–1.95	0.021

Although analysis of the entire cohort did not find an association between TB diagnosis in contacts and index case characteristics (age and HIV status) (Table 6), sub-group analysis among index cases with known HIV status demonstrated a 1.43 fold increased chance of detecting an additional TB case in households known to be HIV-affected households.

**Table 6:** Association of Index Case Characteristics and TB Diagnosis.

Characteristic	OR	95% CI	P Value
**Age group 0–4.99 years**	0.92	0.44–1.92	0.829
(Referent Group: Age group 5–14)			
**Age group ≥15 years**	1.29	0.73–2.26	0.381
**Reported Positive HIV Status**	1.56	0.91–2.65	0.103
(Referent Group: HIV Negative)			
**Reported Unknown HIV Status**	1.31	0.73–2.36	0.359

## Discussion

Case finding activities have long been recommended but not implemented routinely in TB high burden settings [2, 13, 16]. Since 2010, not only have guidelines been released to promote ACF among contacts of TB cases [5] and people living with HIV [4] but competitive funding has become available to support ACF in TB high burden settings [8, 17]. Emerging data now demonstrates that ACF is not only feasible but affords high yield in TB high burden settings [9]. Furthermore, 40 to 80 percent of presumptive and diagnosed TB cases are people living with HIV. Hence, ACF affords a high yield HIV testing platform to help achieve the universal target of diagnosing 90% of all people living with HIV.[18] Unfortunately, limited data is available to inform ACF in children living in TB/HIV-high burden settings [9, 12, 14]. Implemented in a setting with extremely high TB and HIV infection rates, our pediatric-focused contact tracing program demonstrated that the yield of contact tracing is 1.43 fold greater in HIV-affected households. Our findings further estimate a child TB incidence of 1,390 per 100,000 child contacts screened. These findings support current WHO guidelines prioritizing contact tracing of households affected by HIV and including children [12].

The yield of contact tracing is dependent on a number of factors [8, 9]. Yield increases directly as the prevalence of TB increases in the population and the infectivity of the index increases. Our program demonstrated a contact tracing yield of 1.6% for all forms of TB. This was similar to yield reported from other contact tracing programs conducted in TB high-burden settings that included adult and child index cases with all forms of TB; three studies from the Democratic Republic of Congo (2.2%, 2.8%, and 1.3% yield) and one from Pakistan (0.6% yield) [9]. In comparison, research studies have identified TB in 8 to 10% of child contacts in TB high-burden settings, highlighting the potential yield of child TB contact tracing given robust resources[19, 20]. The NNS to find an additional bacteriologically confirmed TB case consistently decreased as the age of the index case and the age of the contacts increased. In contrast, to detect an additional case of any form of TB the NNS was greatest in the 5–14 year old age group, followed by the adults and children under 5 years. This trend likely reflects over diagnosis of bacteriologically unconfirmed, clinically diagnosed TB among young child contacts.

Yield of contact tracing also increases with use of more inclusive eligibility criteria guiding examination of contacts’ sputum [9, 21]. Our program used moderately inclusive criteria as we tested sputum of only contacts who reported one or more TB symptoms; this likely contributes to the higher NNS estimated by our program compared to programs using more inclusive eligibility criteria.

Previous research has been unable to compare the yield of contact tracing originating from child and adult index cases [9] within the same intervention. We demonstrate that the NNS consistently decreased as the age of the index case and the age of the contacts increased for bacteriologically confirmed TB. As the majority of child TB is not bacteriologically confirmed, we garner important information by comparing NNS using all forms of TB as our outcome and demonstrate that the NNS was 40% greater for a child index case compared to an adult index case. Previously reported source case investigations including children hospitalized for TB in TB/HIV high burden settings have demonstrated 2–3% yield [22, 23]. Similarly, the yield of source case investigation originating from index cases under five years of age participating in our program was 2.5%. But, our relatively lower overall program yield of 1.6% likely reflects our intervention’s community based entry point associated with earlier case detection which is associated with a higher NNS [24], lower certainty of diagnosis among children, and realistic implementation of a large community based intervention. Nevertheless, our source case investigations identified 2,476 additional cases for every 100,000 contacts screened suggesting a potential role for source case investigation in TB high burden settings.

Our findings suggest that sputum collection is a predictor of the yield of contact tracing. NNS to detect a bacteriologically confirmed TB case is more than halved in contacts that provided sputum when compared to NNS in contacts that were only symptom screened. We postulate that this association is driven by factors such as advanced disease and older age in the contacts who are able to produce sputum. Regardless of causation, this observation has practical relevance for programmatic implementation of contact tracing and argues for more comprehensive sputum collection among TB contacts.

Our study provides much needed information to apprise contact tracing guidelines [5] in TB/HIV high burden settings. Although previous studies have shown that TB diseased individuals with advanced HIV disease may be less infectious [25, 26], we demonstrated a 1.43 fold increased chance of detecting an additional TB case in households known to be HIV-affected. Consistent with findings of smaller household contact tracing studies conducted within a research platform [19], we demonstrate that in our large interventional program contacts continued to be more likely to have TB disease if they were younger than 5 years of age (OR = 2.01), reported HIV-infection (OR = 4.9), reported one or more TB symptoms (OR = 7.70), or shared a bed with the index case (OR = 1.65). In contrast, contacts with unknown HIV status were less likely to have TB disease (OR = 0.63). We did not find increased risk of TB associated with miners or a death reported in the home. Our findings support current WHO guidelines recommending symptom-based contact screening and prioritization of contact tracing in homes affected by HIV and including children.

The contribution of contact investigation to overall TB case notification in the administrative area of the program varies considerably (range 1% to 14%; pooled estimate 1.8%, 95% CI 0.9–3.6%) [9]. Swaziland has experienced a steady decline in TB incidence due to a number of factors including increased access to antiretroviral therapy for TB patients co-infected with HIV, enhanced integration of TB and HIV chronic care services, improved access to TB diagnosis using new technologies, and increased IPT coverage among people living with HIV [27]. In the first year of program implementation, modeling adjusted for declining national TB notification rates estimated 32% additionality resulting from our contact tracing program. Our program’s additionality declined in subsequent years likely reflecting the identification of prevalent cases in addition to incident cases during initial program implementation [24]. Our findings highlight the importance of robust and long-term program evaluation to accurately assess impact of newly introduced contact tracing interventions.

The impact of any ACF interventions in a TB/HIV high-burden setting is dependent on the program’s ability to initiate preventive and curative TB and HIV treatment among eligible contacts [28]. Although previous studies have demonstrated this potential [22], studies have also demonstrated significant challenges linking new cases to prompt treatment [29] and consistently low uptake of preventive TB treatment [30–35]. Similarly, only 25% of eligible contacts evaluated by our program initiated IPT. Although we did not gather information regarding causation of poor adherence, our previously published work in similar settings demonstrates that adherence may be enhanced by improving available clinic services, correcting misinformation, reducing stigma and providing support to care givers [33, 34]. Our findings highlight the realities of community-based screening interventions and need to allocate adequate resources to support preventive programs and maximize return on investment.

Our study had a number of limitations commonly reported from large-scale contact tracing programs that reflect the challenges of implementing contact tracing in a TB/HIV high burden setting. Rates of HIV positivity have been shown to be equal to or even higher among presumed TB patients than those diagnosed with TB. Although some individuals in our program may have previously completed testing and chosen to not disclose their HIV status to the program team or their families, it is likely that a portion of the 55% of participants with unknown HIV status refused testing. This is a somber reality check of progress needed to achieve the UNAIDS 90-90-90 targets[36, 37]. Among contacts who met criteria for sputum evaluation, 62% provided sputum for evaluation. Although consistent with previous studies that report a range of 13.6–93.4% [9, 38], incomplete sputum evaluation lowers the potential impact of any contact tracing program. The contribution of contact investigation to overall TB notification rate is dependent on the programs coverage rate of index cases. As we were unable to measure our programs coverage rate of index cases, we cannot estimate the full potential of contact tracing in our setting if coverage rate was to increase. Similar to many other contact tracing studies [22, 23, 30], we were unable to follow contacts longitudinally and measure the impact of our intervention on TB prevention.

## Conclusions

Although contact tracing has been recommended as a strategy to increase TB case finding for decades, limited data has been available to inform contact tracing guidelines in children and HIV-affected populations. We demonstrate that contact tracing prioritizing households composed of high numbers of children is not only feasible in a TB/HIV high burden setting but also contributes significantly to overall case detection rates. Our findings provide needed evidence to support existing WHO guidelines prioritizing contact tracing among children and HIV-infected populations in TB high burden settings. Empowering the public health workforce in Swaziland with expertise to screen, rule out and diagnose TB in children would be a worthwhile investment in attaining this. We further demonstrate missed opportunities but true potential to effectively integrate TB and HIV case finding without which it will be impossible to achieve the UNAIDS 90-90-90 targets.

## Supporting Information

S1 File Butimba Family Mapping Tool (FMT). (PDF)
