# Supplementary material for: BUTIMBA: Intensifying the Hunt for Child TB in Swaziland through Household Contact Tracing
Source: PLoS One. 2017 Jan 20;12(1):e0169769. doi: 10.1371/journal.pone.0169769 (PMC5249050; doi:10.1371/journal.pone.0169769)
Supplement: S1 File — (PDF) [file pone.0169769.s001.pdf]

DET Page Number:___________________ **BMU:** __________________ **Baylor EMR :** ___________________ ☐N/A

BUTIMBA: TB REACH FAMILY MAPPING TOOL

Has anyone in the household been employed as a miner? ☐Yes ☐No **Verbal consent obtained for home visit:** ☐Yes ☐No **Date of home visit:**______________

Name of IC: ______________________________________ **IC's TB Register Number:**_________________________ **Date FMT Completed:**___________________

Death in home in prior 2 years: ☐Yes ☐No *(If yes, cause: ☐TB ☐HIV ☐Other illness ☐Accident ☐Other)* **IC's DOB:**___________________ **IC's Sex:** ☐M ☐F

IC's HIV Status: ☐NR ☐R ☐Unk **IC's Phone Number:**______________________

IC's GXP: ☐Not Done ☐MTB Detected ☐MTB Not Detected ("ND") ☐Indeterminate/Invalid *(If MTB Detected: ☐Rif Resistance Detected ☐Rif Resistance NOT Detected)*

IC's Smear: ☐Not Done ☐3+ ☐2+ ☐1+ ☐Scanty ☐Negative (Date Test Result Received: _________________________)

IC's Culture: ☐Not Done ☐Pending ☐Negative ☐Positive (Date Results Rec'd:______________) *(If positive, DST results: Rif* ☐S ☐R */ INH* ☐S ☐R */ Eth* ☐S ☐R */ Strepto* ☐S ☐R*)*

IC's CXR: ☐Not Done ☐Positive ☐Negative/Normal (Date of CXR: _________________________)

Please list all living in the household that are:

Under 5 Years Old

Name and Surname	DOB (DD/MM/YYYY)	Sex		Rel to IC		Sleep Location Compared to IC				TB Screen Questions			TB Scrn		HIV Status			On TB Tx		Ref to Clinic		Sputum Collected					IPT Start Date
		M	F	Child	Other	Same bed	Same room	Same house	Different house	Cough	Fever x ≥2 wks	Poor wt gain/FTT	P	N	R	NR	?	Y	N	Y	N	Yes, at facility	Yes, at home visit	No	If yes, date sputum collected	Tick if GA, NPA or IS	

***Please remember to update TB Screen "Date screening positive" in the DET.*

Update FMT Date: Cough Monitor:

5 - 14 Years Old

Name and Surname	DOB (DD/MM/ YYYY)	Sex		Relation to IC			Sleep Location Compared to IC				TB Screen Questions			TB Scrn		HIV Status			On TB Tx		Ref to Clinic		Sputum Collected					Contact Number
		M	F	Parent/Caregiver	Child	Other	Same bed	Same room	Same house	Different house	Cough	Fever x ≥2 wks	Poor wt gain/FTT	P	N	R	NR	?	Y	N	Y	N	Yes, at facility	Yes, at HV	No	If yes, date sputum collected	Tick if GA, NPA or IS	

***Please remember to update TB Screen “Date screening positive” in the DET.*

15+ Years Old

Name and Surname	DOB (DD/MM/ YYYY)	Sex		Relation to IC			Sleep Location Compared to IC				TB Screen Questions			TB Scrn		HIV Status			On TB Tx		Ref to Clnc		Sputum Collected					Contact Number
		M	F	Parent/Caregiver	Child	Other	Same bed	Same room	Same house	Different house	Cough	Fever x ≥2 wks	Nt Swts x ≥2 wks	P	N	R	NR	?	Y	N	Y	N	Yes, at facility	Yes, at HV	No	If yes, date sputum collected	Tick if GA, NPA or IS	

***Please remember to update TB Screen “Date screening positive” in the DET.*

CM’s Initials: ________ TB Nurse’s Initials: ________

FMT cross-checked with DET: ________ (initials), Date: ________
